# Nightmare disorder and low back pain in veterans: cross-sectional association and effect over time

**DOI:** 10.1093/sleepadvances/zpac030

**Published:** 2022-09-10

**Authors:** Kenneth A Taylor, Skai W Schwartz, Amy C Alman, Adam P Goode, Getachew A Dagne, Yuri V Sebastião, Philip R Foulis

**Affiliations:** Orthopaedic Surgery, Duke University School of Medicine, Durham, NC, USA; Duke Clinical Research Institute, Duke University School of Medicine, Durham, NC, USA; College of Public Health, University of South Florida, Tampa, FL, USA; College of Public Health, University of South Florida, Tampa, FL, USA; Orthopaedic Surgery, Duke University School of Medicine, Durham, NC, USA; Duke Clinical Research Institute, Duke University School of Medicine, Durham, NC, USA; Population Health Sciences, Duke University School of Medicine, Durham, NC, USA; College of Public Health, University of South Florida, Tampa, FL, USA; Division of Global Women’s Health, Department of Obstetrics and Gynecology, School of Medicine, University of North Carolina at Chapel Hill, Chapel Hill, NC, USA; Morsani College of Medicine, University of South Florida, Tampa, FL, USA; Pathology and Laboratory Medicine, James A. Haley Veterans’ Hospital, Tampa, FL, USA

**Keywords:** pain, nightmares, behavioral sleep medicine, epidemiology, psychiatric disorders

## Abstract

Low back pain (LBP) disproportionately impacts US military veterans compared with nonveterans. Although the effect of psychological conditions on LBP is regularly studied, there is little published to date investigating nightmare disorder (NMD) and LBP. The purpose of this study was to (1) investigate whether an association exists between NMD and LBP and (2) estimate the effect of NMD diagnosis on time to LBP. We used a retrospective cohort design with oversampling of those with NMD from the Veterans Health Administration (*n* = 15 983). We used logistic regression to assess for a cross-sectional association between NMD and LBP and survival analysis to estimate the effect of NMD on time to LBP, up to 60-month follow-up, conditioning on age, sex, race, index year, Charlson Comorbidity Index, depression, anxiety, insomnia, combat exposure, and prisoner of war history to address confounding. Odds ratios (with 95% confidence intervals [CIs]) indicated a cross-sectional association of 1.35 (1.13 to 1.60) and 1.21 (1.02 to 1.42) for NMD and LBP within 6 months and 12 months pre- or post-NMD diagnosis, respectively. Hazard ratios (HRs) indicated the effect of NMD on time to LBP that was time-dependent—HR (with 95% CIs) 1.27 (1.02 to 1.59), 1.23 (1.03 to 1.48), 1.19 (1.01 to 1.40), and 1.10 (0.94 to 1.29) in the first 3, 6, 9, and 12 months post-diagnosis, respectively—approximating the null (1.00) at >12 months. The estimated effect of NMD on LBP suggests that improved screening for NMD among veterans may help clinicians and researchers predict (or intervene to reduce) risk of future back pain.

**Statement of Significance**

We used a large electronic health record dataset to investigate the adjusted cross-sectional association of nightmare disorder (NMD) diagnosis with low back pain (LBP). Individuals with NMD had higher odds of having an LBP diagnosis within both 6 and 12 months pre- and post-NMD diagnosis. We also estimated the effect of NMD diagnosis on LBP over time while conditioning on other variables to address confounding. We observed a time-dependent effect, indicating that the risk of LBP is higher within the first year after NMD diagnosis, but this effect estimate approximates the null beyond 12 months. Remaining gaps include the unknown impact of nightmare frequency or NMD descriptors and potential mediators of the estimated effect of NMD on LBP.

## Introduction

Low back pain (LBP) is common and represents the largest cause of years lived with disability worldwide, and has maintained its hold as the leader in this category since the first Global Burden of Disease Study in 1990 [1–4]. Years lived with disability due to LBP has increased over those past three decades [2], resulting in significant financial and economic impacts [5–7]. In the United States, LBP appears to have a disproportionate effect on military veterans, with higher prevalence and severity compared with nonveterans [8].

Among factors contributing to LBP [9], psychological diagnoses (e.g. depression, anxiety, post-traumatic stress disorder) have a notable impact on incident LBP and transitioning to chronicity [9–17]. Similarly, clinical sleep diagnoses such as insomnia and obstructive sleep apnea also impact the incidence and severity of pain conditions through a variety of associated mechanisms (short sleep, sleep fragmentation) [18–24]. One understudied diagnosis when investigating LBP to date that may similarly impact pain outcomes is nightmare disorder (NMD). Two diagnostic criteria for NMD are available that differ slightly, one from the American Academy of Sleep Medicine’s *International Classification of Sleep Disorders*—*Third Edition* (*ICSD-3*) and the other from the American Psychiatric Association’s *Diagnostic and Statistical Manual of Mental Disorders, Fifth Edition* (*DSM-5*). Both describe NMD as (1) repeated with extreme and extended dysphoric dreams that are well-remembered, (2) typically involving threatening scenarios (to physical integrity, security, or survival), (3) marked by rapid alertness and orientation upon waking, and (4) causing impaired function (which may include social and occupational functioning) resulting from clinically significant distress from the repeated experiences [25, 26]. The diagnostic criteria from the American Psychiatric Association go further to include that the nightmares are not attributable to comorbid medical conditions or psychological disorders and that nightmare-related symptoms cannot be explained as the result of substance use or abuse [25].

NMD appears to be relatively rare among the general adult population. The American Academy of Sleep Medicine approximates adult NMD prevalence at 4% [27]; however, studies in other countries have reported reoccurring nightmare prevalence among adults (which may include, but is not limited to NMD) as ranging from 3.5% to 8.3% [28]. Information is limited regarding NMD prevalence among veterans, but a large study of all veterans who sought care through the Veterans Health Administration (VHA) in fiscal years 2000–2010 reported 0.3% prevalence when combining NMD with diagnostic codes for other parasomnias [29]. Exact reasons why this estimate of NMD (plus other parasomnias) prevalence is so low in the VHA study compared with estimates among general adult populations is unclear. This low prevalence estimate may be the result of underreporting of nightmares among veterans and/or differences in age between the VHA and nonveteran adult populations.

While sparse, some studies have established a link between nightmares and LBP. A case–control study of French patients with chronic LBP (101 participants) demonstrated having slept badly at least once a week over the previous months due to nightmares as indicated by the Pittsburgh Sleep Quality Index was more prevalent among individuals with chronic LBP than controls (29.7% vs. 7.5%) [30]. Authors did not present results of their case–control study in terms of odds ratios (ORs), but based on the study’s available information, we were able to calculate an unadjusted OR of 5.2 (95% confidence interval [CI] 2.1 to 12.7), indicating an association between nightmares and chronic LBP. A Finnish study of metal industry workers (902 participants) spanning 28 years also found evidence of nightmares influencing LBP (adjusted for age, sex, and occupational class), with those reporting both sleep disturbances and nightmares (vs. neither) having over a twofold increase in the hazard of first-time hospitalization for any back condition (hazard ratio [HR] 2.4, 95% CI 1.2 to 4.6) and a threefold increase when excluding those who reported chronic or recurrent LBP at baseline (HR 2.9, 95% CI 1.2 to 7.1) [31]. A cross-sectional study of Chinese soldiers (2565 participants) also reported a significant association between LBP and self-reported nightmare frequency, indicating a strong association which increased with increasing nightmare frequency (OR 3.4, 95% CI 2.1 to 5.6) after adjusting for personal and family history of LBP, sleep quality, smoking, and self-perceived fitness [32].

What limited research is available appears to indicate that nightmares may be associated with or influence LBP. We are unaware of any research investigating nightmares (specific to NMD or otherwise) and LBP in any US population. Likewise, all studies discussed above investigating nightmares and LBP utilized self-reported nightmares without considering the additional diagnostic criteria for NMD. As a result, nightmare cases in those studies are likely to reflect a wide variety of conditions associated with dysphoric dream experiences (including, but not limited to NMD) [33]. Administrative data may more accurately reflect NMD diagnostic criteria. Therefore, the purpose of this study was to use available administrative data to (1) investigate whether an association exists between NMD and LBP and (2) estimate the distinct effect of NMD on time to LBP.

## Methods

### Study design

Using retrospective observational cohort data collected from the James A. Haley Veterans’ Hospital (JAHVH) in Tampa, FL, we conducted two analyses: (1) a cross-sectional analysis aimed at assessing association between NMD and LBP and (2) a target trial emulation to estimate the distinct effect of NMD on time to LBP. The Target Trial Framework specifies the features of a pragmatic randomized trial that would estimate (in this case) the effect of NMD on time to LBP (Supplementary Table S1) [34, 35]. Such a randomized trial would be unethical to perform even if it were feasible; therefore, emulation of the target trial using observational data remains the only way to estimate this effect. Luckily, when target trials are successfully emulated with observational data, estimated effects are the same as they would be in the specified target trial [35, 36]. The Institutional Review Boards at the University of South Florida and JAHVH approved this study for the initial data collection. Subsequent addendums were filed and approved the use of this previously collected data for this study. We collected data in two parts to obtain the original sample. One part of our data collection focused on obtaining a sample without NMD. To that end, we used an algorithmic program to select one random date in each quarter per year from first-quarter 2007 through fourth-quarter 2011, totaling 20 dates. We used these to create a list from which to sample comprising all individuals having an encounter at JAHVH or any of its associated clinics on the randomly selected dates. A trial-and-error method was used to adjust the number of individuals selected from each quarter, since it was possible for an individual to be listed from more than one quarter, until a final random sample of 22 000 individuals with an equal number from each quarter was achieved. In the second part of our data collection, we identified individuals with NMD diagnosis (our exposure of interest) using International Classification of Diseases, Ninth Revision (ICD-9) code 307.47. Due to concerns about the rarity of this diagnosis [29], we oversampled on those with the exposure of interest. All individuals having at least one nightmare-related encounter from 2007 through 2011 at the JAHVH or its associated clinics were included in the original NMD case sample. (The prevalence of NMD in this study was higher than would typically be observed in the VHA because of this oversampling of those individuals with NMD.) All electronic health record data were collected from 2006 to 2016 up to the date of electronic health record collection (June 8, 2016) for both the exposed (NMD diagnosis) and unexposed groups.

### Participants

After the original sample was obtained, index date was set for all individuals. For those with NMD, index date was set at the first date of NMD diagnosis. Individuals who were sampled twice (i.e. sampled as part of the unexposed group despite having NMD) were removed from the unexposed group before assigning index dates for the unexposed group. Index date for those without NMD was assigned to a random visit date between 2006 and 2011, frequency matching for index year compared with the group with NMD.

After determining index year, individuals were excluded if they had the following at or at any time before index date: cancers other than primary prostate or skin cancer, spinal surgery or procedure, myelopathy, cauda equina syndrome, spinal cord injury, vertebral fracture or dislocation, congenital or acquired deformity of the spine, spinal infection, inflammatory spinal disease, or osseous disease or deficit. Individuals were further excluded from our final sample if they had encounters related to pregnancy or transportation accidents at or within 12 months before index date. These exclusions were determined using available ICD-9 and International Classification of Diseases, Tenth Revision (ICD-10) codes based on recommendations published regarding identifying individuals with spinal pain and relevant exclusion criteria with administrative data (see Supplementary Table S2) [37]. The recommendations applied included requiring at least two ICD-9 or ICD-10 codes to indicate exclusion to prevent unnecessary exclusions from a single code being used as a diagnosis of suspicion rather than a confirmed exclusionary diagnosis. Single ICD-9 and ICD-10 codes were used to identify exclusions only when determining pregnancy, spinal procedures or surgeries, and transportation accidents. Individuals were also excluded if they were determined to be hospital personnel rather than a patient or if they had inconsistent data (e.g. death date listed before index date). Individuals with a diagnosis of post-traumatic stress disorder (ICD-9 309.81 or ICD-10 F43.11, F43.12) [38, 39] at or before index date were removed from this sample because we aimed to investigate NMD as an independent diagnosis rather than as a symptom of post-traumatic stress disorder. Individuals with missing data for any of the variables adjusted for in our analyses were excluded via listwise deletion.

### Variables and measurement

Available electronic health record information was used to determine demographic information for each individual: age, sex, race, combat exposure, and prisoner of war history. Both combat exposure and prisoner of war history were available only as dichotomous variables without additional detail. We used available SAS macros to calculate the Charlson Comorbidity Index to account for overall health status using ICD-9 and ICD-10 codes from the earliest available record up to 12 months after index date [40–45]. Depression and anxiety diagnoses at or before index date was determined based on the presence of at least one ICD-9 (296.2, 296.20–296.25, 296.3, 296.30–296.35, 300.4, 311 and 300.0, 300.2, respectively) or ICD-10 codes (F32, F32.0–F32.9, F33, F33.0–F33.9, F34.1, F34.2 and F40, F41, respectively) associated with these diagnoses [46]. Insomnia diagnosis at or before index date was also determined based on the presence of at least one ICD-9 (780.52) or ICD-10 code (G47.0) associated with insomnia diagnosis [47].

LBP diagnosis (with or without associated leg pain) was determined using the ICD-9 and ICD-10 codes listed in Supplementary Table S3. Although the use of administrative data to identify episodes of LBP may miss some transient episodes that occur without a visit to a healthcare provider, it will catch episodes that are bothersome enough to result in consultation with a healthcare provider. Time to LBP or censoring event (defined below) was calculated as the difference in days from index date to the event.

### Censoring

Participants were censored if LBP was not observed before the first of three potential censoring events: date of death from any cause, loss to follow-up, or administrative end of follow-up for the study. If participants were primarily seen at clinics associated with the Orlando Veterans Affairs Medical Center, which at one time was considered part of the JAHVH system, then they were considered lost to follow-up and censored on November 24, 2009 (assuming death or LBP event did not occur first). This date represents when the Orlando clinics separated from the JAHVH system, which included separation of electronic medical records after that date. Administrative censoring occurred after 60 months of follow-up if no other censoring events or LBP were observed before that time.

### Statistical power

We performed power analysis before the primary statistical analysis based on the available sample, as the available data were already collected for a previous, unpublished study. Power analysis was performed in Stata/SE 16 (College Station, TX) to obtain power estimates for a range of HR values based on the available number of individuals in the exposed and unexposed groups after exclusions and assuming a rate of 25% cumulative LBP events at various time points. The results of this power analysis are shown in Supplementary Figure S1 and Supplementary Table S4. Results indicate at least 79% power to detect HR values of ≥1.2 at ≤4 years and at least 72% power to detect HR values of ≥1.2 by the end of study follow-up.

### Statistical methods

**Figure 1.**
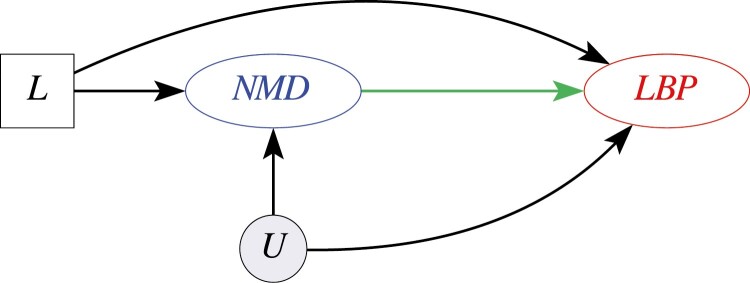
Simplified Directed Acyclic Graph of the Effect of Nightmare Disorder Diagnosis on Low Back Pain. Nightmare disorder (NMD) diagnosis is the exposure of interest and low back pain (LBP) is the outcome of interest. The edge from NMD to LBP represents our effect of interest in this study. The node labeled ‘L’ represents measured confounders that we conditioned on in our analysis. The node labeled ‘U’ represents potential confounding that is unmeasured and unknown.

All other statistical analyses were performed using SAS 9.4 (Cary, NC). First, we performed logistic regression to determine if there was an association between NMD and LBP in this population with the outcomes of LBP within 6 months pre- or post-index date and within 12 months pre- or post-index date. Performing this initial cross-sectional analysis allowed for comparison of effect size of NMD diagnosis in this population to that in the study of Chinese soldiers [32]. Second, we used Cox proportional hazards modeling to estimate the effect of NMD on time to LBP encounter (see Directed Acyclic Graphs in Figure 1 and Supplementary Figure S2). While cross-sectional and longitudinal analyses included some overlapping time periods, these analyses differed in their treatment of time. In cross-sectional analyses, we used a single time window (within either 6- or 12-months) which did not differentiate LBP events occurring pre- or post-NMD diagnosis. In contrast, the longitudinal analyses specifically model time to LBP event post-NMD diagnosis. Because prior bouts of LBP are common, our primary longitudinal analysis included individuals with and without a history of LBP events before baseline. Finally, sensitivity analyses were performed excluding all individuals who had LBP within the 6 months and 12 months before index date to remove individuals with LBP encounters after index date that may have been the result of an ongoing episode of LBP and those with chronic (lasting ≥3 months) [48] or recurrent LBP. While the definition of recurrent LBP differs across published literature, a 12-month period of exclusion before index date meets most of these definitions. Because of how common the experience of prior LBP is and the impact of excluding large proportions of the total sample on power, a 6-month period of exclusion before index date was also used [49]. All statistical modeling in this study was adjusted for age, sex, race, index year, Charlson Comorbidity Index, combat exposure, depression, anxiety, insomnia, and prisoner of war status to address confounding.

## Results

### Participants

**Figure 2.**
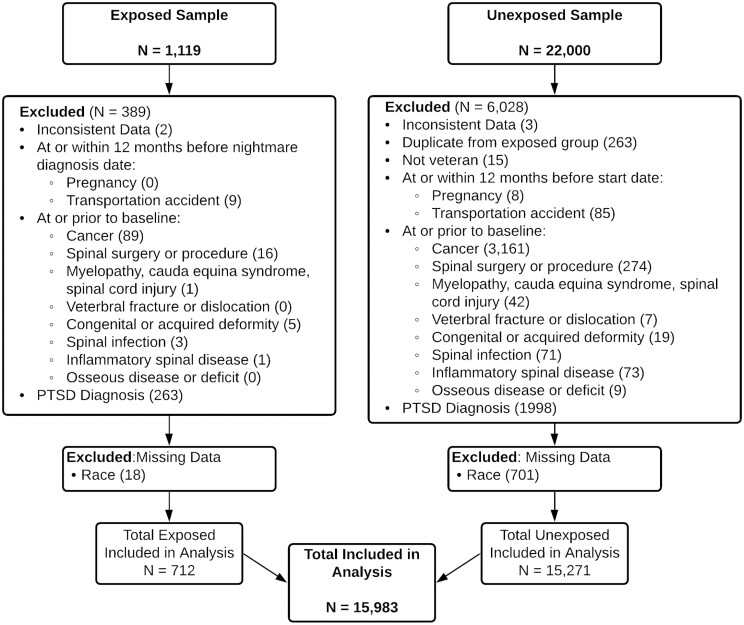
Study flow diagram.

The final sample included in analyses comprised 15 983 individuals (712 with NMD) after applying exclusion criteria and listwise deletion of those with missing confounder data (see Figure 2). Participant characteristics are presented in Table 1 by NMD status and by LBP status by the end of follow-up. Of those with NMD diagnosis, 63.6% were associated with visits to clinics or clinicians specializing in mental health (27.4%), sleep (24.4%), pulmonology (2.2%), neurology (1.1%), or specific post-9/11 deployment (6.3%) or polytrauma programs that include psychological evaluation by a mental health professional.

**Table 1.** Participant characteristics at index date by exposure and outcome status, Tampa, FL, 2006–2016^*^

	Nightmare disorder		Low back pain event	
	Yes (*N* = 730)	No (*N* = 15 972)	Yes (*N* = 5772)	No (*N* = 10 930)
Age, mean (*SD*)	55.3 (15.42)	62.0 (14.96)	59.2 (14.31)	63.1 (15.25)
Female	64 (8.8%)	1345 (8.4%)	552 (9.6%)	857 (7.8%)
Race				
Black	81 (11.4%)	1859 (12.2%)	785 (13.9%)	1155 (11.2%)
White	565 (79.4%)	12 075 (79.1%)	4376 (77.3%)	8264 (80.0%)
Other	66 (9.3%)	1337 (8.8%)	498 (8.8%)	905 (8.8%)
Missing	18	701	113	606
Charlson Comorbidity Index, mean (*SD*)	1.3 (1.89)	1.7 (2.05)	1.7 (2.11)	1.6 (2.01)
Depression	159 (21.8%)	1688 (10.6%)	628 (10.8%)	577 (5.3%)
Anxiety	84 (11.5%)	11 118 (7.0%)	882 (15.3%)	965 (8.8%)
Insomnia	159 (21.8%)	609 (3.8%)	314 (5.4%)	373 (3.4%)
Combat exposure	67 (9.2%)	1415 (8.9%)	511 (8.9%)	971 (8.9%)
Prisoner of war survivor	2 (0.3%)	42 (0.3%)	11 (0.2%)	33 (0.3%)
Index year				
2006	46 (6.3%)	1123 (7.0%)	303 (5.2%)	866 (7.9%)
2007	244 (33.4%)	5150 (32.2%)	1567 (27.1%)	3827 (35.0%)
2008	172 (23.6%)	3924 (24.6%)	1435 (24.9%)	2661 (24.3%)
2009	117 (16.0%)	2771 (17.3%)	1165 (20.2%)	1723 (15.8%)
2010	64 (8.8%)	1362 (8.5%)	573 (9.9%)	853 (7.8%)
2011	87 (11.9%)	1642 (10.3%)	729 (12.6%)	1000 (9.1%)
Nightmare disorder	—	—	262 (4.5%)	468 (4.3%)

^*^Data are *n* (%) except where indicated.

### Cross-sectional association between NMD and LBP

The OR for NMD with the outcome of LBP within the 6 months pre- or post-index date was 1.35 (95% CI 1.13 to 1.60). The OR for NMD when expanding this window to include LBP within 12 months pre- or post-index date was 1.21 (95% CI 1.02 to 1.42).

### Effect of NMD on time to LBP

**Figure 3.**
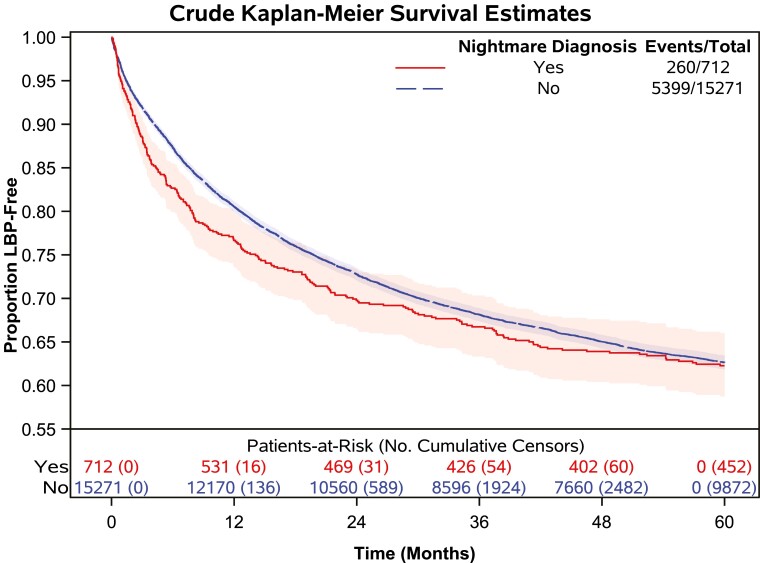
Crude Kaplan-Meier survival estimates.

**Figure 4.**
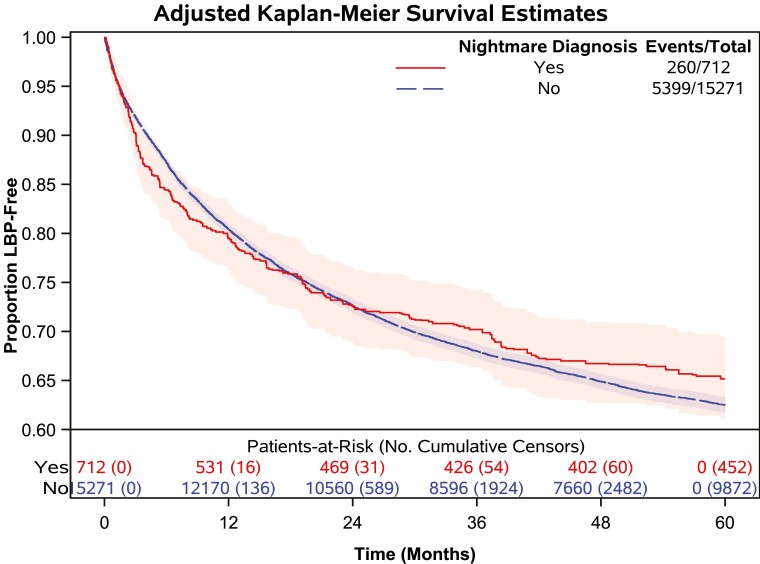
Adjusted Kaplan-Meier survival estimates.

In total 51 780.18 person-years of follow-up (median follow-up: 4.09 person-years) were observed in the final sample. The cumulative incidence of first LBP encounters during the observed 5-year follow-up time was 35.4% (5659 participants). Cumulative LBP events and censoring events by exposure are presented in Table 2 along with mean and 25th percentile survival (LBP-free) time and median follow-up time. Crude and adjusted Kaplan-Meier survival curves stratified by NMD are presented in Figures 3 and 4, respectively. The adjusted Kaplan-Meier survival curves indicate that there was a violation of the proportional hazards assumption when adjusting for potential confounders. Upon further investigation, Schoenfeld residuals for NMD from the adjusted model were correlated with observed follow-up time, confirming that the proportional hazards assumption was violated. To correct this violation, we included an NMD-by-time interaction term in the model [50]. The HRs for the effect of NMD on time to LBP are presented in Table 3 with different time-point cutoffs, as are results from sensitivity analyses. The HR for the effect of NMD on LBP at baseline was 1.15 (95% CI 0.97 to 1.37). The point estimate of this effect increased to 1.27 (95% CI 1.02 to 1.59) at 3 months and gradually decreased over time. Beyond 12 months (HR 1.10; 95% CI 0.94 to 1.29) the estimated effect size continued to decrease toward the null.

**Table 2.** Measures of low back pain-free survival time, observed events, and censoring by exposure

	Nightmare disorder	
	Yes (*N* = 712)	No (*N* = 15 271)
Median follow-up time, years	4.94	4.03
Survival time, years (95% CI)^*^		
Mean	3.51 (3.36 to 3.66)	3.65 (3.50 to 3.80)
25th percentile	1.17 (0.79 to 1.64)	1.65 (1.54 to 1.75)
Cumulative events, *n* (%)		
Low back pain	260 (36.5%)	5399 (34.4%)
Censor cause, *n* (%)		
Administrative	383 (53.8%)	6960 (45.6%)
Orlando VAMC exit	31 (4.4%)	1318 (8.6%)
Death	38 (5.3%)	1594 (10.4%)

VAMC, Veterans Affairs Medical Center.

^*^Crude estimates.

**Table 3.** Estimated effect of nightmare disorder on time to low back pain over 5-year follow-up^*^

Time from index, months	Primary analysis		6-Month sensitivity analysis^†^		12-Month sensitivity analysis^‡^	
	Estimate	95% Confidence interval	Estimate	95% Confidence interval	Estimate	95% Confidence interval
0	1.15	0.97 to 1.37	1.04	0.80 to 1.36	0.98	0.73 to 1.32
3	1.27	1.02 to 1.59	1.54	1.06 to 1.74	1.34	0.85 to 2.10
6	1.23	1.03 to 1.48	1.30	0.97 to 1.74	1.23	0.88 to 1.73
9	1.19	1.01 to 1.40	1.13	0.87 to 1.47	1.07	0.79 to 1.44
12	1.10	0.94 to 1.29	1.02	0.80 to 1.29	0.96	0.73 to 1.26
24	1.03	0.89 to 1.18	0.88	0.72 to 1.08	0.85	0.68 to 1.06
36	0.97	0.85 to 1.11	0.83	0.69 to 1.00	0.80	0.65 to 0.98
48	0.96	0.84 to 1.09	0.82	0.69 to 0.98	0.79	0.66 to 0.96
60	0.94	0.83 to 1.06	0.80	0.67 to 0.95	0.77	0.64 to 0.92

^*^All estimates and associated confidence intervals are hazard ratios adjusted for age, sex, race, depression, anxiety, insomnia, combat exposure, prisoner of war status, index year, and Charlson Comorbidity Index. Estimate at time zero is the estimated effect of nightmare disorder diagnosis at time of diagnosis.

^†^ *N* = 13 721 (564 with nightmare disorder); 2262 excluded due to prior LBP (148 with nightmare disorder; 1946 with low back pain outcome after index date).

^‡^ *N* = 13 189 (533 with nightmare disorder); 2794 excluded due to prior LBP (179 with nightmare disorder; 2329 with low back pain outcome after index date).

Sensitivity analyses demonstrated similar point estimates to the primary analysis, with largest effect seen at 3 months when excluding individuals with LBP in encounters in the prior 6 months (HR 1.54; 95% CI 1.06 to 1.74) and prior 12 months (HR 1.34; 95% CI 0.85 to 2.10). Like in the primary analysis, point estimates receded toward the null as time continued. Later time points (2 years and beyond) in sensitivity analyses had point estimates that were notably on the other side of the null (<1.00).

## Discussion

Few studies have looked at the relationship between nightmares and LBP, and ours is the first study to the best of our knowledge to investigate this relationship utilizing a large sample of electronic health record data and the first to investigate NMD and LBP in a US-based cohort. Our results indicated an overall association between NMD and LBP, with the adjusted odds of LBP being 1.45 and 1.30 times higher among people with NMD within 6 and 12 months of that initial diagnosis of NMD, respectively. This adjusted cross-sectional association may indicate either a bidirectional association between NMD and LBP or the effect of NMD being present for months before the individual having an initial health encounter where NMD was diagnosed. The second goal of our study was to estimate the effect of NMD on time to LBP, with NMD preceding LBP. Our effect estimates demonstrated a time-dependent effect of NMD on time to LBP, with the effect being larger at times closer to NMD diagnosis and gradually decreasing toward the null over the observed follow-up period. The exact reason for this time-dependent effect is unclear. We speculate that the decreasing strength of this effect over time could be related to the fact that chronic NMD (persisting ≥6 months) [25] is rare, and there is at least some evidence that NMD can be successfully treated through minimal contact with healthcare providers for intervention [28]. This means that the duration of NMD is unlikely to persist beyond 6 months after onset assuming it has been identified early, and it makes intuitive sense that any effect of NMD on LBP would fade as NMD symptoms improve as well. However, other explanations for this observed change over time may exist that we cannot confidently rule out in this study.

Sensitivity analyses indicated similar effect point estimates in the 12 months after NMD diagnosis when removing those with previous LBP within 6 and 12 months of their index date, albeit with less precise estimates that included the null value in most cases. This loss of estimate precision in our sensitivity analyses is at least partly because we removed 34.4% and 41.2% of the cumulative LBP events in the survival analysis by removing those with prior LBP in the 6 and 12 months before their index date, respectively. We expected that removal of such a large proportion of events would negatively impact variance and statistical power. Point estimates consistently moved to the other side of the null across all analyses at some point beyond 12 months, with NMD appearing protective at the furthest time points (3–5 years). However, this is likely because those with NMD more frequently had an LBP event before 3 years of follow-up. Our analysis did not allow for the consideration of future repeated events after the first LBP event following the index date. Therefore, individuals with NMD who have not yet had a first post-NMD LBP event by those later time periods were less likely to have an LBP event at those times.

The observed effect size of NMD in our study was small compared to the effect of other psychological diagnoses on LBP [13–17]. However, the estimated effect of NMD consistently peaked early post-NMD diagnosis across longitudinal analyses, with point estimates indicating 1.27- to 1.54-fold increase in instantaneous risk at 3-months. These results indicate that even though NMD may be a relatively rare diagnosis, it has a notable short-term impact on LBP that, to date, has not been identified or considered in clinical practice and research. The observed effect sizes in our study were smaller than the large effects seen in other studies investigating nightmares (regardless of NMD status) and LBP presented in the introduction [30–32]. There are two possible reasons for this, both of which are related to the use of administrative data to determine NMD diagnosis status. First, all studies of nightmares and LBP mentioned earlier used self-report for nightmares that only considered the presence of dysphoric dreams without consideration for the additional NMD diagnostic criteria used by physicians and/or psychologists. The use of an ICD-9 code instead of self-report of dysphoric dreams alone to determine NMD diagnosis is more likely to reflect the *DSM-5* and *ICSD-3* diagnostic criteria [25, 26].

Second, while the observed effect size of NMD on LBP was relatively small, it is likely that our effect estimates are biased toward the null. NMD is often left unreported by patients experiencing the condition, with the majority either deciding not to actively seek clinical care or being unaware that treatment options are available [28]. Additionally, actively screening for nightmares in the absence of other psychological conditions is rare [28]. This decision not to report ongoing nightmares from the patient and failure to screen for their presence by the healthcare system almost certainly leads to some misclassification bias. This underdiagnosis described above is unlikely to depend on LBP diagnosis, increasing the likelihood of bias toward the null and suppressed estimates of the effect of NMD. Estimates may be further biased due to misclassification of LBP, as those attending visits with other concerns or being seen more frequently for other conditions are more likely to have transient LBP detected and recorded in their electronic health record than those with only transient LBP. In most cases it is likely that the outcome in this study represents LBP that is bothersome enough for the individual to seek care and most transient LBP is left undetected, based on our use of ICD-9 and ICD-10 codes from electronic health record data to determine the LBP outcome. Therefore, it seems reasonable to assume that the effect of NMD on LBP may be larger than observed in our results; our results are a conservative estimate of the effect of NMD on LBP.

The previously discussed study that investigated the relationship between several risk factors and LBP among a cohort of Chinese soldiers found that the odds of LBP increased significantly as the frequency of nightmares within the past 5 months increased [32]. Unfortunately, the available administrative data in our study regarding NMD did not include details of severity (i.e. frequency of nightmares during the week) [25], which prevented us from further exploring whether the observed effect sizes in our results varied based on the frequency of nightmares. Although recently developed and yet to be validated against the gold standard for NMD diagnosis (structured clinical interview) [51], incorporation of the Nightmare Disorder Index may be a potential solution for future research to improve the accuracy of self-report in identifying NMD cases based on *DSM-5* criteria and collect details regarding severity [52].

There are additional limitations to our study. First, our results may not generalize well to the broader population as our study involved US military veterans—a group that is currently predominantly white, older, and male. Second, the use of administrative data introduces multiple additional limitations beyond those already discussed, as there are at least two recognized diagnostic criteria for NMD and these two sets of criteria do not perfectly match [25, 26]. It is unclear based on the available administrative data which diagnostic criteria were utilized to make the NMD diagnosis or to what degree either criteria were adhered to for diagnosis. Since the two criteria are associated with professional medical specialty associations (psychiatry and sleep medicine), it is possible that both diagnostic criteria are reflected since our collection of the exposed group data was not limited to one specific medical specialty. However, we excluded individuals with PTSD and conditioned on anxiety, depression, and insomnia to limit confounding from other diagnoses to match more closely the more restrictive of the two criteria. We did not have more detailed sleep information available (e.g. sleep duration and excessive daytime sleepiness) to condition on due to the administrative nature of our data. Third, the outcome of interest was LBP regardless of the presence of associated leg pain. Previously published work indicates that comorbidities may affect LBP differently depending on whether associated leg pain is present, and it is possible that an NMD may also affect time to LBP differently depending on LBP type [53]. Finally, censoring participants may lead to potential selection bias that could bias toward or away from the null depending on its presence and magnitude [54].

In summary, ours is the first study to the best of our knowledge to investigate the effect of NMD diagnosis on LBP using administrative data with a large, US-based sample. Our results indicate an estimated effect of NMD on LBP that is time-dependent, initially increasing the risk of LBP, peaking at 3 months, and decaying as time since initial diagnosis increases. These results suggest that improved screening for NMD among veterans may aide clinicians and researchers in predicting an individual’s short-term risk for future LBP events. Further, our results suggest that prevention and/or successful early intervention may limit the effect of NMD on LBP. Future research regarding this effect should aim to gather additional information regarding nightmare frequency, use new tools (such as the Nightmare Disorder Index) in combination with gold standard diagnosis and/or administrative data to reduce potential for misclassification bias, and attempt to clarify the exact mechanisms underlying the observed effect.

## Supplementary Material

zpac030_suppl_Supplementary_Material

## Data Availability

To protect the privacy and protected health information of individuals that participated in the study the data underlying this article cannot be shared publicly. The data will be shared on reasonable request to the corresponding author.
