# Supplementary material for: Nightmare disorder and low back pain in veterans: cross-sectional association and effect over time
Source: Sleep Adv. 2022 Sep 10;3(1):zpac030. doi: 10.1093/sleepadvances/zpac030 (PMC9648406; doi:10.1093/sleepadvances/zpac030)
Supplement: zpac030_suppl_Supplementary_Material [file zpac030_suppl_supplementary_material.docx]

**TITLE**: Nightmare Disorder and Low Back Pain in Veterans: Cross-Sectional Association and Effect Over Time

**Authors**: Kenneth A. Taylor, DPT, PhD^1,2^; Skai W. Schwartz, PhD^3^; Amy C. Alman, PhD^3^; Adam P. Goode, DPT, PhD^1,2,4^; Getachew A. Dagne, PhD^3^; Yuri V. Sebastião, PhD^5^; Philip R. Foulis, MD, MPH^6,7^

^1^Duke University, Orthopaedic Surgery, Durham, NC, USA; ^2^Duke University, Duke Clinical Research Institute, Durham, NC, USA; ^3^University of South Florida, College of Public Health, Tampa, FL, USA; ^4^Duke University, Population Health Sciences, Durham, NC, USA; ^5^University of North Carolina at Chapel Hill, School of Medicine, Department of Obstetrics and Gynecology, Division of Global Women’s Health, Chapel Hill, NC, USA; ^6^University of South Florida, Morsani College of Medicine, Tampa, FL, USA; ^7^James A. Haley Veterans’ Hospital, Pathology and Laboratory Medicine, Tampa, FL, USA

**Corresponding Author:** Kenneth A. Taylor, DPT, PhD, Duke Clinical Research Institute, 300 West Morgan Street, Durham, NC 27701, US. Phone: 765-437-1624; Fax: 919-684-1846. E-mail: [kenneth.taylor@duke.edu](mailto:kenneth.taylor@duke.edu)

**Supplemental Content**

Table S1. Target Trial Framework for Estimating the Effect of Nightmare Disorder on Low Back Pain

| **Protocol Component** | **Description** | **Specified Target Trial** | **Observational Emulation** |
| --- | --- | --- | --- |
| **Eligibility Criteria** | Who will be included in the study? | Veterans enrolled in the veterans health administration in Tampa, FL | Same as specification |
| **Treatment Strategies** | What interventions will eligible persons receive? | 1. Nightmare disorder  2. No nightmare disorder | Same as for specification |
| **Treatment Assignment** | How will eligible persons be assigned to the interventions? | Eligible persons randomly assigned to exposure and aware of exposure assignment | Eligible persons assigned to the exposure with which their data were compatible at the time of eligibility/enrollment |
| **Outcomes** | What outcomes in eligible persons will be compared among intervention groups? | Time to low back pain diagnosis at subsequent visit | Same as for specification.  *Required data: electronic health record with ICD-9/ICD-10 codes and dates* |
| **Follow-up** | During which period will eligible persons be followed in the study? | From treatment assignment until loss to follow-up, administrative end of follow-up, or death; whichever occurs first. | Same as for specification.  *Required data:* *date of loss to follow-up and death* |
| **Causal Estimand** | Which counterfactual contrasts will be estimated using the above data? | Intention-to-treat effect (effect of being assigned to treatment)  Per-protocol effect (effect of receiving treatment as indicated in protocol) | Observational analog of per-protocol effect |
| **Statistical Analysis** | How will the counterfactual contrasts be estimated? | Intention-to-treat analysis  Per-protocol analysis (requires adjustment for pre-assignment confounding) | Same as per-protocol analysis  *Required data: pre-assignment confounding variables* |

Table S2. Diagnosis and Procedure Codes Used to Identify Exclusion Criteria

| **Exclusion** | **ICD-9** | **ICD-10** |
| --- | --- | --- |
| Cancer | 140.xx – 172.xx, 174.xx – 184.xx, 186.xx – 239.xx, V10.1x – V10.45, V10.47 – V10.81, V10.84 – V10.91 | C00.xxx – C42.xxx, C45.xxx – C60.xxx, C62.xxx – D49.xxx, O9A.1xx, Z85.0xx – Z85.45, Z85.47 – Z85.819, Z85.83x – Z86.00x |
| Lumbar region myelopathy, cauda equina syndrome, or spinal cord injury | 344.6, 344.60, 344.61, 721.4, 721.42, 722.73 | G83.4, M47.15, M47.16, M51.05, M51.06, M51.07, S34.xxxx |
| Lumbar region spinal fracture or dislocation | 805.4, 805.5, 805.6, 805.7, 806.4, 806.5, 806.6, 806.7, 839.2, 839.20, 839.3, 839.30, 839.41, 839.42, 839.51, 839.52 | M43.5X5 – M43.5X7, M48.4 – M48.48, M48.55 – M48.58, M53.2X5 – M53.2X8, S32.0-S32.2, S32.9, S33.1 – S33.4 |
| Congenital or acquired lumbar region spinal deformity | 756.1, 756.10 – 756.19 | M40.05, M40.15, M40.205, M40.45 – M40.47, M40.55 – M40.57, M41.05 – M41.07, M41.115 – M41.117, M41.125 – M41.127, M41.25 – M41.27, M41.35, M41.45 – M41.47, M41.55 – M41.57, M41.85 – M41.87, M43.25 – M43.28, M43.8X5 – M43.8X8, Q76.415, Q76.425, Q786.427, Q76.428 |
| Infection | 324.1, 324.9, 730.xx | G06.1, M46.25 – M46.28, M46.35 – M46.38, M46.45 – M46.48, M46.55 – M46.58, M54.04 – M54.08, M86.xxx |
| Inflammatory spinal disease | 720.xx | M46.05 – M46.08, M46.1, M46.85 – M46.88, M46.95 – M46.98, M45.5 – M45.8, M48.8X5 – M48.8X8, M49.85 – M49.88 |
| Pregnancy | 630.xx – 679.xx | O00.xxx – O99.xxx |
| Osseous disease or deficit | 731.xx, V82.21 | M89.75x |
| Spinal surgery or procedure | 03.0x, 03.02x, 03.09x, 03.2x, 03.21, 03.29, 03.6x, 80.5x, 81.0x, 81.3x, 81.6x, 722.83 | M96.1xx-M96.5xx, M96.65x |
|  | *CPT Codes:*  008Wxxx, 008Xxxx, 008Yxxx, 220xxxx – 228xxxx, 6626xxx – 6229xxx, 623xxxx – 6371xx | |
| Transportation accident | E800.x – E849.x | V00.xxxx – V99.xxxx |

Abbreviations: CPT, Current Procedural Terminology; ICD-9, International Classification of Diseases, Ninth Revision; ICD-10, International Classification of Diseases, Tenth Revision.

Table S3. Diagnosis Codes Used to Identify Low Back Pain Outcome

| **Region** | **ICD-9** | **ICD-10** |
| --- | --- | --- |
| Thoraco-lumbar spine | *Local Low Back Pain*:  722.5, 722.51  *Low Back Pain with Leg Pain*:  722.1, 724.02 | *Local Low Back Pain*:  M43.15, M46.45, M51.34, M51.35, M51.45, M53.85  *Low Back Pain with Leg Pain*:  M48.05, M51.2, M51.25, M54.15 |
| Lumbar spine | *Local Low Back Pain*:  722.32, 722.93, 724.2, 739.3, 847.2  *Low Back Pain with Leg Pain*:  721.10, 722.10, 724.3 | *Local Low Back Pain*:  M43.16, M46.46, M51.36, M51.46, M51.86, M53.86, M54.5, M99.03, M99.83, S33.4xxx  *Low Back Pain with Leg Pain*:  M48.06, M48.061, M51.26, M51.27, M54.16, M54.3, M54.30, M54.31, M54.32, M54.4, M54.40, M54.41, M54.42 |
| Lumbosacral spine | *Local Low Back Pain*:  721.3, 722.52, 739.4, 846, 846.0, 846.1, 846.2, 846.3, 846.8, 846.9 | *Local Low Back Pain*:  M43.17, M46.47, M47.817, M51.37, M51.87, M53.87, M54.47, S33.6xxx, S33.8xxx, S33.9xxx  *Low Back Pain with Leg Pain*:  M48.07, M54.17 |
| Sacrococcygeal spine | *Local Low Back Pain*:  724.6, 724.7, 724.70, 724.71, 724.79, 847.3, 847.4 | *Local Low Back Pain*:  M43.18, M46.48, M53.3, M53.88, M99.04, M99.84  *Low Back Pain with Leg Pain*:  M48.08, M54.18 |
| Abbreviations: ICD-9, International Classification of Diseases, Ninth Revision; ICD-10, International Classification of Diseases, Tenth Revision. | | |

Figure S1. Estimated Power for Two-Sample Comparison of Survivor Functions


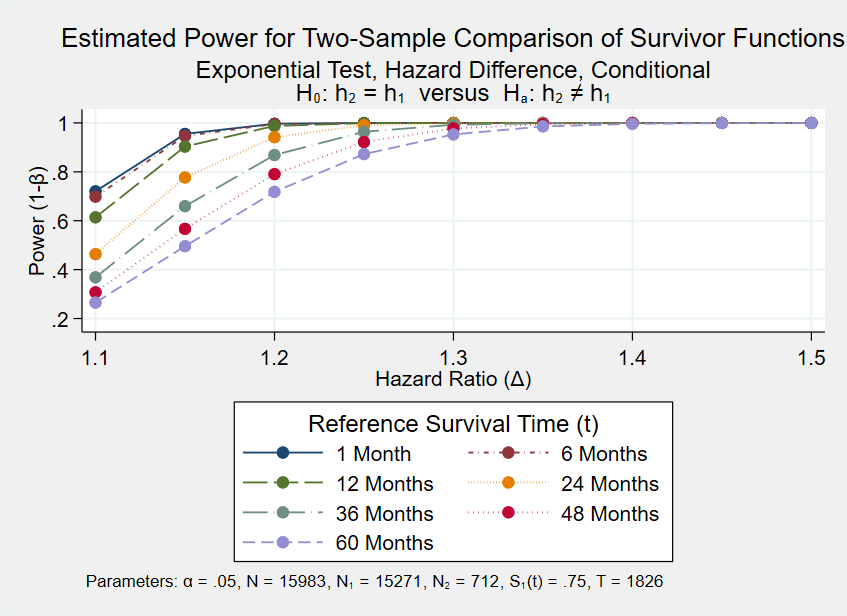


Table S4. Estimated Power for Two-Sample Comparison of Survivor Functions

**Figure S2.** Detailed Directed Acyclic Graph of the Effect of Nightmare Disorder Diagnosis on Low Back Pain.


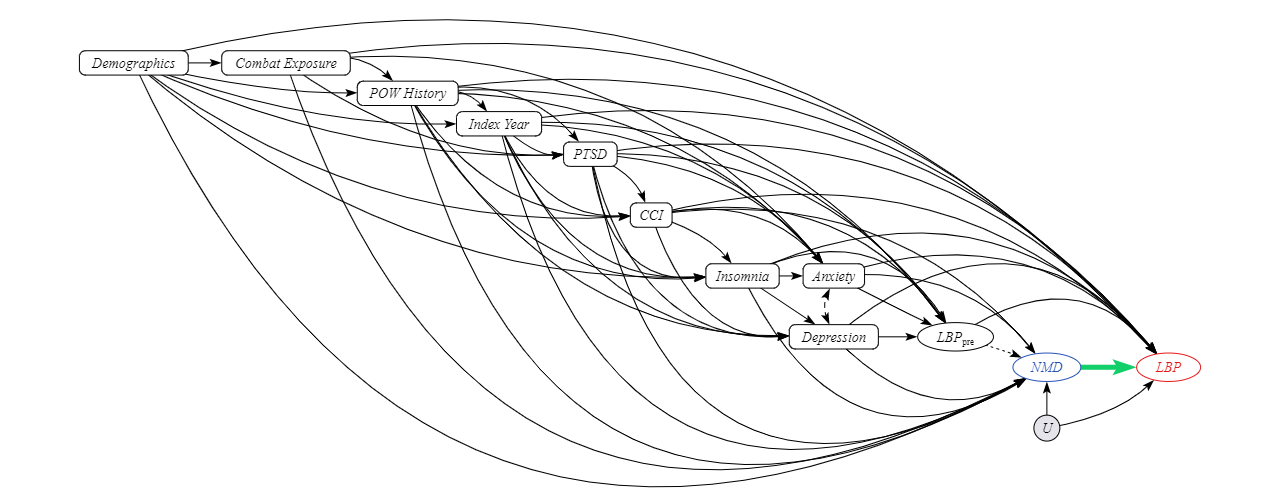


Nightmare disorder (NMD) diagnosis is the exposure of interest and low back pain (LBP) is the outcome of interest. The edge from NMD to LBP represents our effect of interest in this study. Nodes in rectangular boxes represent confounders of the effect of interest that were conditioned on in analysis. LBP_pre_ represents LBP in the 12 months prior to NMD or index date, which was conditioned on in sensitivity analysis. The dotted edge from LBP_pre_ to NMD represents the uncertain effect of LBP_pre_ on future NMD (and therefore uncertainty about whether it meets the structural definition of a confounder). The dashed edge from anxiety tot depression represents the uncertain temporal order of these two in relation to one another. The node labeled ‘U’ represents potential confounding that is unmeasured and unknown. Other abbreviations: CCI = Charlson Comorbidity Index; POW = Prisoner of War; PTSD = Post-Traumatic Stress Disorder.
